# Prenatal diagnosis of Wolf-Hirschhorn syndrome confirmed by comparative genomic hybridization array: report of two cases and review of the literature

**DOI:** 10.1186/1755-8166-5-12

**Published:** 2012-02-28

**Authors:** Stavros Sifakis, Emmanouil Manolakos, Annalisa Vetro, Dimitra Kappou, Panagiotis Peitsidis, Maria Kontodiou, Antonios Garas, Nikolaos Vrachnis, Anastasia Konstandinidou, Orsetta Zuffardi, Sandro Orru, Ioannis Papoulidis

**Affiliations:** 1 Department of Obstetrics & Gynecology, University of Crete, Heraklion, Greece; 2 Eurogenetica S.A., Laboratory of Genetics, Athens-Thessaloniki, Greece; 3 Dipartimento di Patologia Umana ed Ereditaria, Universita di Pavia, Pavia, Italia; 4 Helena Venizelou Hospital, Athens, Greece; 5 Department of Obstetrics & Gynecology, University of Thessalia, Larissa, Greece; 6 2nd Department of Obstetrics and Gynecology, Aretaieion Hospital, Univeristy of Athens, Athens, Greece; 7 1st Department of Pathology, Univeristy of Athens, Athens, Greece; 8 Cattedra di Genetica Medica, Universita di Cagliari, Cagliari, Italia

**Keywords:** 4p- syndrome, Comparative genomic hybridization array, "Greek warrior" helmet profile, Fluorescent situ hybridization, Prenatal diagnosis, Wolf-Hirschhorn syndrome

## Abstract

Wolf-Hirschhorn syndrome (WHS) is a well known genetic condition caused by a partial deletion of the short arm of chromosome 4. The great variability in the extent of the 4p deletion and the possible contribution of additional genetic rearrangements lead to a wide spectrum of clinical manifestations. The majority of the reports of prenatally diagnosed WHS cases are associated with large 4p deletions identified by conventional chromosome analysis; however, the widespread clinical use of novel molecular techniques such as array comparative genomic hybridization (a-CGH) has increased the detection rate of submicroscopic chromosomal aberrations associated with WHS phenotype. We provide a report of two fetuses with WHS presenting with intrauterine growth restriction as an isolated finding or combined with oligohydramnios and abnormal Doppler waveform in umbilical artery and uterine arteries. Standard karyotyping demonstrated a deletion on chromosome 4 in both cases [del(4)(p15.33) and del(4)(p15.31), respectively] and further application of a-CGH confirmed the diagnosis and offered a precise characterization of the genetic defect. A detailed review of the currently available literature on the prenatal diagnostic approach of WHS in terms of fetal sonographic assessment and molecular cytogenetic investigation is also provided.

## Background

Wolf-Hirschhorn syndrome (WHS; OMIM 194190) [1], also known as deletion 4p and 4p-syndrome, is a well known clinical condition caused by a partial deletion of the short arm of chromosome 4. WHS was first (and independently) described by Wolf et al. (1965) and Hirschhorn et al. (1965) [2,3]; thereafter, more than 180 documented cases have been published in the literature, most of them diagnosed postnatally. The prevalence of WHS is reported to be around 1/50.000 live births with a 2:1 female/male ratio; however, this is likely underestimated because of under-recognition or misdiagnosis of affected individuals [4,5].

In the majority of cases, WHS is caused by a "pure" deletion of 4p16 with no other cytogenetic abnormality while in the remaining cases, there could be a more complicated cytogenetic finding such as chromosome 4 ring, 4p- mosaicism, or a derivative chromosome 4 resulting from either a de novo or inherited unbalanced translocation [5,6]. The complexity of the WHS-associated basic genomic changes is an important factor explaining phenotypic variability; though the typical clinical features include growth restriction of prenatal onset, profound psychomotor retardation, seizures, skeletal abnormalities, and a distinctive facial appearance [7]. Associated major malformations with variable incidence (30-70%) are mainly related to midline fusion defects such as midline scalp defects, agenesis of corpus callosum, cleft lip/palate, heart defects, and urinary tract malformations [7,8].

Most prenatally diagnosed cases of WHS are associated with large 4p deletions identified by conventional chromosome analysis while the widespread clinical use of novel high-resolution molecular techniques such as array comparative genomic hybridization (a-CGH) increased the detection rate of submicroscopic chromosomal aberrations that could also lead to a WHS phenotype. Herein, we present two WHS cases suspected upon abnormal signs in prenatal ultrasonography, diagnosed with conventional cytogenetics and further characterized through a-CGH. A detailed review of the current literature on prenatal diagnosis of WHS is also provided.

## Cases presentation

### Case 1

A 25-year-old primigravida was referred to our clinic at 23 weeks of gestation due to fetal intrauterine growth restriction (IUGR). The family history was unremarkable and first-trimester screening test for chromosomal aneuploidies was normal. Ultrasound examination showed fetal measurements (BPD, HC, AC, FL) below the 5^th ^centile, consistent with severe symmetrical IUGR. Umbilical artery Doppler flow velocimetry exhibited reverse end-diastolic flow and pulsatility index (PI) was 1.83 (>95^th ^centile); in addition, uterine artery PI were bilaterally increased (>95^th ^centile) measuring 2.25 and 1.78 respectively and notches were present as well. No fetal malformation was present. As the amniotic fluid volume was reduced (AFI < 5), the ultrasound imaging of fetal facial anomalies was hampered. Upon abnormal ultrasound findings, an amniocentesis was performed and karyotype analysis led to the diagnosis of WHS which was further confirmed by a-CGH and FISH. After genetic counseling, termination of pregnancy was performed at parents' request at 25 weeks of gestation. A male neonate was delivered vaginally after medical induction with prostaglandins. Detailed pathological examination of the proband was denied by the parents.

### Case 2

**Figure 1:**
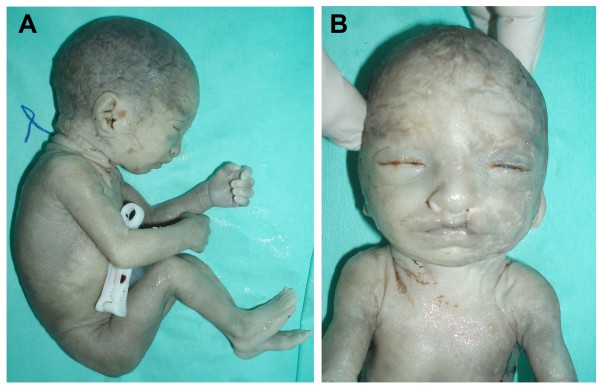
**Autopsy of a 24 weeks' gestation female fetus after pregnancy termination (Case 2) that showed external features of facial dysmorphism with bilateral cleft lip, hypertelorism, broad and high nasal bridge, small filter and large ears**.

A 37-year-old primigravida was referred to our clinic for genetic counseling at 23 weeks of gestation due to presence of growth restriction in serial obstetric scans since the 13^th ^week of gestation. The couple was healthy, no consanguineous, with unremarkable medical history. An amniocentesis was performed at 23 weeks of pregnancy, and the fetal karyotype was compatible with the diagnosis of WHS. a-CGH analysis showed with high precision a 19.3 Mb terminal 4p deletion, in the area 4p15.3-pter. After extensive counseling, the family decided to terminate the pregnancy and agreed to an autopsy for the fetus. A female fetus was delivered at 24 weeks after medical induction. Fetal autopsy showed external features of facial dysmorphism with bilateral cleft lip, hypertelorism, broad and high nasal bridge, small filter and large ears (Figure 1). The skull was oval shaped, consistent with the helmet-like typical description of WHS related facial appearance. The somatometric parameters indicated a symmetric restriction of fetal growth. Organ dissection showed a small cerebellum with neuroglial heterotopias, a cardiac defect (patent foramen ovale), intestinal malrotation, hypoplastic kidneys, accessory spleen and enlarged ovaries. Placenta was hypotrophic with a weight of 170 g without any significant macroscopic or histological abnormalities; the umbilical cord presented three vessels. Growth velocities were equivalent to 20 weeks of pregnancy.

### Cytogenetic and molecular cytogenetics analysis

Amniotic fluid was collected from case 1 and case 2 at 23 weeks of gestation. Cytogenetic analysis was performed on cultured amniocytes by G-banding according to standard procedures. At least 20 metaphases were analyzed per case, revealing a male karyotype with terminal deletion of the short arm of one of chromosome 4 [46, XY, del(4)(p15.33)] in case 1, and a female karyotype with terminal deletion of the short arm of one of chromosome 4 [46, XY, del(4)(p15.31)] in case 2. a-CGH was done on DNA from cultured amniocytes to characterize the extent of the deletions using a 100 kb resolution array (kit 44 K) in case 1 and a 40 kb resolution (kit 180 K) in case 2. Molecular karyotyping was carried out through oligonucleotide array-CGH platforms (Agilent Technologies, Santa Clara, CA) as described elsewhere [9].

**Figure 2:**
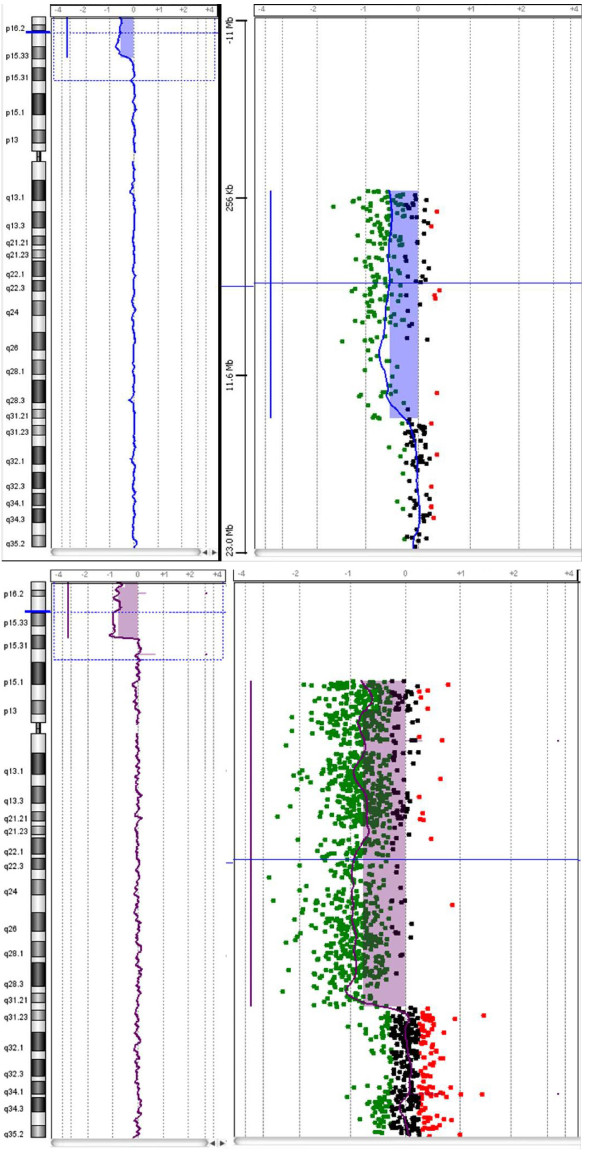
**a. a-CGH profile of chromosome 4 showing an terminal deletion**. To the left, the whole chromosome 4 view. To the right, the enlarged view of the rearrangement as provided by Agilent Technologies, CGH Analytics 3.5.14. The overall size of the deletion was about 14.7 Mb. **b**. a-CGH profile of chromosome 4 showing a terminal deletion. To the left, the whole chromosome 4 view. To the right, the enlarged view of the rearrangement as provided by Agilent Technologies, CGH Analytics 3.5.14. The overall size of the deletion was about 19.3 Mb.

In case 1 the 14.7 Mb deletion, involved the cytobands from p15.33 to pter (first probe on the 44 K array at 62,447 bp, deleted), having its proximal breakpoint between 14,678,744 bp and 14,744,566 bp (Figure 2a). In case 2 the 19.3 deletion involved the cytobands from p15.31 to 4pter (first probe on the 180 K array at 35,882 bp, deleted) having its proximal breakpoint between 19,341,751 bp and 19,364,876 bp (Figure 2b). The positions of oligomers refer to the Human Genome March 2006 (versions NCBI 36, hg18) assembly.

Cultured amniocytes were subjected to fluorescent in situ hybridization (FISH) for further confirmation of the diagnosis. The subtelomeric FISH was performed by using the commercially available TelVysion 4p Spectrum Green probe following the manufacturer's instructions (Vysis Inc, Downers Grove, Ill, USA). In both cases the probe showed a signal on only one homologue. Parental karyotyping was found to be normal. Hybridization with the probe on metaphase chromosomes of the parents showed normal signal on both chromosomes 4 and neither parent was found to carry a translocation of the 4pter region (data not shown).

## Discussion

We report two cases of WHS presented with IUGR as an isolated finding or combined with fetal and uterine arteries Doppler abnormalities and oligohydramnios. After invasive testing, conventional cytogenetic investigation led to a diagnosis of WHS; in addition, molecular analysis of the cultured amniocytes with a-CGH and FISH further defined the precise breakpoints of the two deletions.

WHS is a well-described multiple congenital anomaly and mental retardation syndrome caused by partial deletion of the short arm of chromosome 4 involving at least a 165 kb segment of 4p16.3 [7,10,11]. Prenatal diagnosis of WHS is usually confirmed by detection of a cytogenetically visible 4p- deletion discovered after invasive testing performed because of advanced maternal age, severe IUGR (which is the most frequent ultrasound finding, associated or not with other fetal abnormalities), or known parental balanced chromosomal rearrangement [12-19]. In case 1, IUGR was further complicated by Doppler abnormalities in the umbilical artery, bilaterally increased uterine artery PI and oligohydramnios, whereas in case 2, early onset growth restriction was not accompanied with abnormal Doppler or decreased amniotic fluid volume. The ultrasonographic presentation of WHS with IUGR and a notch on the uterine artery also overlap with previously described case by Levaillant et al. [15], while oligohydramnios as a unique finding or associated with other fetal malformations has also been reported in fetuses with WHS [20-24]. A wide range of other anatomical abnormalitites as renal hypoplasia, skeletal dysplasias, hypospadias, diaphragmatic hernia, single umbilical artery also complicates these cases with variable incidence [7,25-31]. In addition, craniofacial dysmorphic features such as microcephaly, "Greek warrior helmet" profile (the broad high nasal bridge continuing to the forehead), prominent glabella, high arched eyebrows and hypertelorism are strongly evocative of WHS [7,26,32,33].

Unfortunately, minor anatomical defects indicative of facial dysmorphism in our case 2 were missed by serial ultrasound scans between 13 and 22 weeks. Several reports of concomitant WHS and other structural chromosomal aberrations as a result of an unbalanced translocation display complex phenotypes and confuse some of the correlations [33-35]. A brief overview of the ultrasound features, the mode of the cytogenetic analysis applied and the perinatal outcome in 36 WHS cases, including our 2 cases and 34 other published cases, is presented in Additional file 1: Table S1. In the context of growth retardation, a reference ultrasonography with 2-D and 3-D fetal facial imaging and/or a detailed prenatal fetal brain evaluation with CT/MRI analysis and fetal echocardiography could be helpful in adding clues towards diagnosis and orientate karyotype analysis on 4p- extremities [15,36,37].

Regarding the molecular basis of WHS, in about 55% of cases, WHS results from an isolated 4p deletion (a so-called "'pure deletion"); about 40-45% of affected individuals have an unbalanced translocation (de novo or inherited from a familial balanced rearrangement) characterized by both a deletion of 4p and a partial trisomy of a different chromosome arm; in the remaining cases, there is a complex rearrangement leading to a 4p16.3 deletion (e.g., chromosome 4 ring) [38,39]. Unbalanced translocations involving the short arms of chromosomes 4 and 8 appear with high frequency in several large series of WHS patients [40-43]. These rearrangements usually arise as a result of a) a homologous non-allelic recombination mediated by olfactory receptors (OR)-gene clusters in both 4p and 8p, or b) a parental inversion polymorphism on 4p16 [44,45]. Recent studies point to a multigenic profile of WHS that contributes to the complex phenotype though two critical regions (WHSCR1 and -2) have been implicated in the pathogenesis of the syndrome [8,46,47]. WHSCR-1 is a 165 kb area approximately 2 Mb from the telomere of 4p and includes the WHS candidate gene 2 (*WHSC2*) and part of *WSHC1 *which is implicated in growth delay and facial characteristics [48-50]. *WHSCR-2*, that contributes to the basic phenotype (typical craniofacial pattern, mild mental retardation, growth delay and seizures) resides in a 300-600 kb interval positioned between 1.9 and 1.6-1.3 Mb from the telomere and is contiguous and telomeric to *WHSCR-1*. This genomic region includes a third critical gene called *LETM1 *(leucine zipper/EF-hand-containing transmembrane) associated with the neuromuscular features of WHS patients such as seizures disorders [5,51], and partially the *WHSC1*. Moreover, recent studies suggest that the fibroblast growth factor receptor-like 1 (*FGFRL1) *represents a plausible candidate gene for part of the craniofacial phenotype of WHS [47,52].

An increasing number of genotype-phenotype correlation studies compare specific clinical features of patients with different sized 4p deletions in order to refine the 4p phenotypic map but the variable expressivity or penetrance of the clinical features and the fact that WHS is likely to be a contiguous gene syndrome, makes it a challenging task. According to a recent study WHS cases can be divided into three clinical categories: the first one comprises a microdeletion not exceeding 3.5 Mb at 4p16-4pter results in a mild phenotype and is likely to be under diagnosed, the second one is associated to deletions between 5 and 18 Mb that present with severe psychomotor delay and typical abnormalities whereas those greater than 22 Mb at 4p15-4pter consist the third category associated with major malformations [8]. However, Battaglia et al. (2001) demonstrated that a submicroscopic deletion that was detected only by FISH may account for the severe WHS phenotype and concluded that there is no such a strict correlation [53]. Alternative mechanisms that can lead to complex phenotypes include: a) unbalanced translocation mutations resulting in 4p deletion and partial trisomy affecting the final phenotype; b) allelic variation in the homologous 4p region; c) mutations in modifier genes located outside the deleted regions; d) position effects and telomere silencing; e) different genetic background and post-zygotic mutational events [43,46]. Differential diagnosis of WHS should include the proximal interstitial 4p deletion which is a discrete syndrome that usually involves bands 4p12-p16 that are proximal to and exclude the WHS critical region [54].

The majority of prenatally diagnosed cases of WHS reported in the medical literature are delineated by conventional cytogenetic analysis, but during the last decade the availability of new technologies especially a-CGH have enabled a more precise description of the molecular mechanisms that can account for the WHS phenotype [23,55]. Indeed, few published reports refer to cases that a standard karyotype was interpreted as normal and a required subsequent molecular analysis by FISH or/and a-CGH upon prenatally or postnatally identified fetal malformations allowed the final diagnosis [11,33,37,56]. A prenatal misdiagnosis of a WHS case is more likely when the fetus presents only with fetal growth restriction or other non-specific or minor features and the standard karyotype results to be balanced [15,23,36,37,57]. Conventional G-banded cytogenetic analysis seems to detect approximately 50-60% of WHS cases while application of FISH analysis using a WHSCR probe detects more that 95% of deletions in WHS [39,53]. In addition, a-CGH can detect all currently known deletions of the WHSCR and determine if the deletion is "pure" or part of a more complex imbalance more accurately than either FISH or conventional G-band analysis alone [39]. A comprehensive analysis of the role of a-CGH in the evaluation of WHS patients demonstrated that the true prevalence of unbalanced translocations is certainly higher than reported previously and is approximately 45% as both karyotype and routine FISH analysis of the region may not detect these cases; also a-CGH adds information on approximate size of both the deletion and duplication compared to a subtelomeric FISH assay [43]. Although both of our cases were associated with cytogenetically visible deletions, we applied a-CGH analysis to confirm that they were pure distal deletions, to define their extent at molecular level and to establish a firm diagnosis. We also applied FISH analysis to further confirm our findings, to extend the investigation to both couples and define the potential presence of a balanced rearrangement involving 4p16.3 in the parents of a proband so as to provide a thorough genetic counseling. In conclusion, growth restriction as an isolated finding or associated with facial dysmorphism and/or other major malformations such as renal or skeletal abnormalities and midline fusion defects may be indicative of a WHS case and should trigger cytogenetic investigation. A combined diagnostic approach based on conventional karyotyping and molecular analysis, would offer a definitive result within the time frame required for management of the affected pregnancy and for a prompt genetic counseling about the long term complications and poor prognosis of these cases. This is crucial as, according to the data presented in the Additional file 1: Table S1, most of the parents opt for pregnancy termination. Furthermore, as part of the genetic counseling prenatal testing should be offered to families in which one parent is known to be a carrier of a chromosome rearrangement involving 4p16.3 Additional investigation with high-resolution techniques such as a-CGH is nowadays strongly recommended particularly in case of discordance between prenatal ultrasound findings and normal karyotype. In the future, the implementation of this technique in the routine practice of prenatal diagnosis will improve the diagnostic yield in pregnancies with abnormal ultrasound findings and particularly to WHS, it will enable a more precise estimation of the true incidence of the syndrome and will advance our knowledge regarding the genotype-phenotype correlations.

## Competing interests

The authors declare that they have no competing interests.

## Authors' contributions

SS and EM drafted the manuscript and coordinate the whole project. EM, AV, MK, OZ, SO, IP performed cytogenetic analysis, a-CGH, and FISH. SS, AG, NV performed the clinical evaluation of the pregnancies. DK and PP participated in the coordination and helped to draft the manuscript. AK performed the pathological examination and helped to draft the manuscript. All authors have read and approved the manuscript.

## Consent

Written informed consent was obtained from the parents for publication of these Case reports and any accompanying images. A copy of the written consent is available for review by the Editor-in-Chief of this journal.

## Supplementary Material

Additional file 1 **Table S1**. Reported cases of prenatal diagnosis of WHS: sonographic findings, karyotype, and pregnancy outcome. Click here for file
