# Supplementary material for: Prenatal diagnosis of Wolf-Hirschhorn syndrome confirmed by comparative genomic hybridization array: report of two cases and review of the literature
Source: Mol Cytogenet. 2012 Feb 28;5:12. doi: 10.1186/1755-8166-5-12 (PMC3307480; doi:10.1186/1755-8166-5-12)
Supplement: Additional file 1 — Table S1. Reported cases of prenatal diagnosis of WHS: sonographic findings, karyotype, and pregnancy outcome. [file 1755-8166-5-12-S1.DOC]

**Additional file 1: Table S1.** Reported cases of prenatal diagnosis of WHS: sonographic findings, karyotype, and pregnancy outcome.

| **Authors & Year** | **GW at diagnosis** | **Ultrasonographic findings** | **Cytogenetic method** | **Karyotype** | **Pregnancy outcome** |
| --- | --- | --- | --- | --- | --- |
| Blunt et al, 1977 [12] | 17 | No reported; investigation due to advanced maternal age | G-banding | 46,XX,del(4)(p?) | TOP |
| Vamos et al,1985 [20] | 20 | oligohydramnios | G-banding | 46,XX,der(4),t(4;20)(p16;p12), inv(18) (p11q11)pat | TOP |
| Eiben et al, 1988 [16] | 26 | IUGR, cleft lip, esophageal atresia | G-banding | 46,XY,del(4)(p15.2) | TOP |
| Verloes et al, 1991 [17] | 24 | IUGR, cystic hygroma, retrognathism, hypertelorism, univentricular heart, atrial septal defects, chorioangioma | G-banding | 46,XY,del(4)(p14) | TOP |
| Tachdjan et al, 1992 [18] | 26 | IUGR, cleft lip and palate, renal hypoplasia | G-banding | 46,XY,del(4)(p16) | TOP |
|  | 33 | IUGR, diaphragmatic hernia, SUA | G-banding | 46,XY,del(4)(p16) | TOP |
|  | 26 | IUGR | G-banding | 46,XX,del(4)(p14) | TOP |
|  | 33 | IUGR | G-banding | 46,XX,del(4)(p16) | TOP |
| Vinals et al, 1994 [25] | 29 | IUGR, hypospadias | G-banding | 46,XY,del(4)(p14) | Live birth at 37w;  at 8 mo severe psychomotor delay |
| Phelan et al, 1995 [34] | 16 | No reported; investigation due to advanced maternal age | G-banding | 47,XX,+21/ 47,XX,del(4)(p16),+21 | Stillbirth at 34w |
| Chen et al, 1998 [19] | 27 | IUGR, cardiomegaly with arrythmia, microcephaly, asymmetry of upper limps, thick nuchal fold, ventricular septal defects | G-banding | 45,XX,der(4)t(4;14)(p16.3;q12),-14 | TOP |
| Sergi et al., 1998 [30] | 30 | IUGR, fetal distress | G-banding | 46,XX,del(4) (pter-->13) | Neonatal death due to respiratory insufficiency |
| Kohlschmidt et al, 2000 [13] | 20 | No reported; investigation due to paternal balanced chromosomal translocation | G-banding | 46,XY,der(4)t(4;18)(p15.32;p11.21)pat | TOP |
| Petek et al, 2000 [21] | 32 | IUGR, anhydramnios | G-banding, FISH | 46,XY,add(4)(p16.3)  ish dup(4)(q26qter) | Stillbirth at 39w |
| Schinzel 2000, [31] | 31 | IUGR, hydronephrosis | G-banding | 46,XX,del(4)(p16.3) | TOP |
|  | 32 | IUGR, olihydramnios,  cystic kindney | G-banding | 46 XX,der(4)t(4;13)(p14;q11) | TOP |
| Witters et al, 2001 [22] | 21 | CPC, oligohydramnios | G-banding,  FISH | 45,XX,-4,+t(4;21)(p16.3;q11),-21 | TOP |
| De Keersmaecker  et al, 2002 [36] | 34 | IUGR, CPC, periventricular cysts, low set ears, hyperechogenic bowel | G-banding,  FISH | 46,XX,del(4)(p16) | TOP |
| Tapper et al, 2002 [35] | 17 | Cystic hygroma, clubbed feet, SUA, bilateral cleft lip, lumbar meningomyelocele, cervical hemivertebrae, left pleural effusion, left diaphragmatic hernia, ventricular septal defect | G-banding, FISH | 46,XX,der(4)t(4;13)(p16;q32) | TOP |
| Aslan et al, 2003 [14] | 29 | IUGR, deformity of lower limp, undescended testes, hypospadias | G-banding, FISH | 46,XY,del(4)(p14) | TOP |
| Dietze et al., 2004 [26] | 27 | IUGR, flat profile, clubfeet, polyhydramnios | G-banding, FISH | 46,XX,del(4)(p16) | TOP |
| Boog et al, 2004 [37] | 32 | IUGR, flat profile, thick prefrontal skin, retrognathism | G-banding | 46,XN,del(4)(p16.3) | TOP |
|  | 16 | IUGR | G-banding | 46,XN,del(4)(p16.3) | TOP |
|  | 33 | IUGR, ascites, septated cerebral ventricular system | G-banding; FISH at 18 mo of age | 46,XX  ish del(4)(p16.3) | Live birth of a retarded neonate at 39w; severe developmental delay and epilepsy at 18 mo |
|  | 35 | Bilateral CPC, retrognathism, frontal horns ventriculomegaly, bilateral renal hypoplasia, placental chorioangioma | G-banding, secondary FISH | 46,XX  ish del(4)(p16.3) | Live birth of a retarded neonate; facial dysmorphism |
|  | 31 | IUGR, microcephaly, corpus callosum agenesis, interhemispheric cerebral cyst, cleft palate, microretrognathia, hypertelorism, low-set ears with pretragial tumors, absent gallbladder, bilateral renal hypoplasia | G-banding | 46,XN,del(4)(p16) | TOP |
|  | 23 | Increased NT, IUGR, ventricular septal defect, SUA, club feet | Normal karyotype, secondary FISH | 46,XN ish del(4)(p16.3p16.3)(WHS-) | TOP |
| Levaillant et al, 2005 [15] | 19 | IUGR, Greek warrior helmet sign | G-banding, FISH | 46,XX,del(4)(p15.3)  ish del(4)(p16.3)(WHS-) | TOP |
| Sase et al, 2005 [32] | 33 | IUGR, facial dysmorphism (high forehead, microretrognathia, prominent glabella, short philtrum) | G-banding, FISH | 46,XX,del(4)(p15.2) | TOP |
| Beaujard et al, 2005 [27] | 33 | IUGR, microcephaly, cleft lip, bilateral renal hypoplasia | G-banding, FISH | 46,XX,der(4)(:p14->p16.1::p16.1->qter). | TOP |
| Cassacia et al, 2006 [28] | 22 | Mild growth restriction, left-sided diaphragmatic hernia | G-banding, FISH | 46,XX ish del(4)(p16.3) | Live birth of a SGA neonate at 38w; developmental delay |
| Basgul et al, 2006 [24] | 27 | IUGR, oligohydramnios, left-sided diaphragmatic hernia, cystic hygroma | G-banding, FISH | 46,XX,del(4)(p15.2) | TOP |
| Chao et al, 2006 [23] | 23 | Oligohydramnios | G-banding,  a-CGH | 46,XY,der(4)t(4;13)(p15.3;p11.2)pat  Array: del(4)(p15.31) | TOP |
| Chen et al, 2008 [33] | 30 | IUGR, Greek helmet facial profile, SUA, prominent glabella, “lobster-claw’’ deformities of hands and feet | a-CGH | 46,XY,der(4)t(4;10)(p16.1;q25.1)pat | Premature delivery at 32w, WHS facial profile, split hand-foot malformation, neonatal death. |
| Current Case 1 | 23 | IUGR, abnormal umbilical artery Doppler | G-banding, FISH,a-CGH | 46,XY,del(4)(p15.33) | TOP |
| Current Case 2 | 24 | IUGR | G-banding, FISH,a-CGH | 46,XΧ,del(4)(p15.31) | TOP |

CPC: choroid plexus cyst; GW: gestational week; IUGR: intrauterine growth retardation; SGA: small for gestational age; SUA: single umbilical artery; TOP: termination of pregnancy.
